# Distinguishing ‘dual’ from ‘duplicated’ right coronary artery: Revisiting the nomenclature

**DOI:** 10.34172/jcvtr.2023.31753

**Published:** 2023-09-23

**Authors:** Niraj Nirmal Pandey, Aprateem Mukherjee, Nitish Naik, Priya Jagia

**Affiliations:** ^1^Department of Cardiovascular Radiology and Endovascular Interventions, All India Institute of Medical Sciences, New Delhi, India; ^2^Department of Cardiology, All India Institute of Medical Sciences, New Delhi, India

**Keywords:** Coronary artery disease, Computed tomography angiography, Cardiac-gated imaging techniques

## Abstract

We report a case of a 53-year-old man with a "short RCA" seen coursing within the proximal part of the right atrioventricular (AV) groove and terminating in the mid-portion of the right AV groove and a "long RCA" seen to have a proximal course outside the right AV groove, over the free wall of the right ventricle, where it gave rise to the right ventricular and acute marginal branches before returning to the right AV groove in its distal course. The discussion highlights the need for revisiting the nomenclature of "dual RCA and drawing a distinction between "dual" and "duplicated" RCA.

## Case History

**Figure 1:**
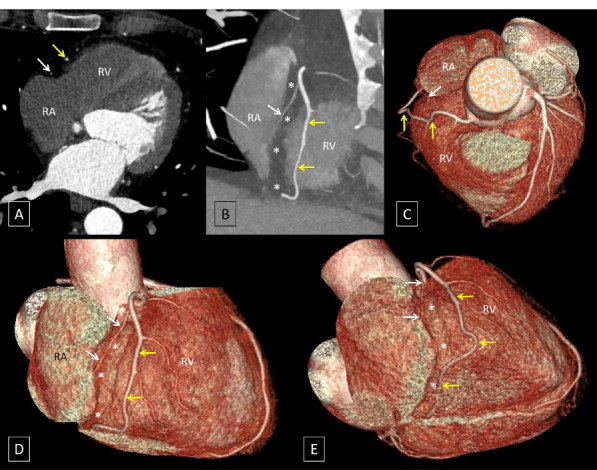
Oblique axial** (A), **oblique coronal** (B) **and volume rendered images** (C to E) **demonstrate the RCA proper dividing into two branches. The posterior branch (white arrows) courses within the proximal part of the right atrioventricular (AV) groove (indicated by asterisks) and terminates in the mid-portion of the right AV groove. The anterior branch (yellow arrows) has a proximal course outside the right AV groove, over the free wall of the right ventricle (RV), where it gave rise to the right ventricular and acute marginal branches before returning to the right AV groove in its distal course. [RA: right atrium]

 A 53-year-old man with complaints of atypical chest pain underwent coronary CT angiography to rule out atherosclerotic coronary artery disease (CAD). While no obstructive CAD was seen, an interesting variation in right coronary artery (RCA) morphology was incidentally detected. After origin of the conal artery, the RCA proper was seen dividing into two segments. The posterior branch or the “short RCA” was seen coursing within the proximal part of the right atrioventricular (AV) groove and terminated in the mid-portion of the right AV groove. The anterior branch or the “long RCA” was seen to have a proximal course outside the right AV groove, over the free wall of the right ventricle, where it gave rise to the right ventricular and acute marginal branches before returning to the right AV groove in its distal course (Figure 1).

## Discussion

**Figure 2:**
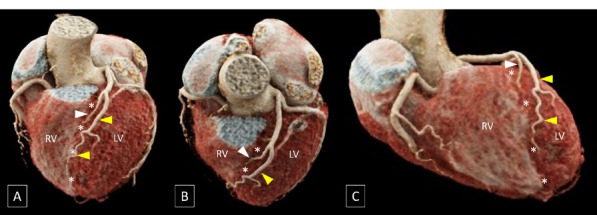
Volume rendered images** (A to C) **show a short left anterior descending artery (LAD, white arrowhead) which terminates in the proximal part of the anterior interventricular groove (indicated by asterisks) and a long LAD (yellow arrowheads), that initially courses outside the anterior interventricular groove on the left ventricular (LV) side and re-enters the groove in its distal part. [RV: right ventricle]

**Figure 3:**
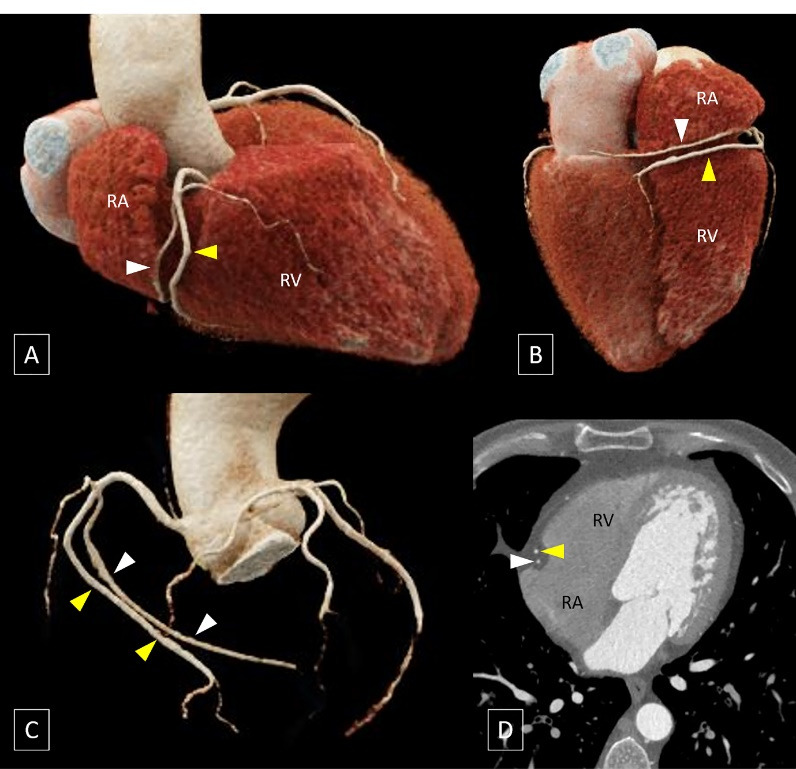
Volume rendered images** (A to C) **and oblique coronal image** (D) **showsthe presence of two parallel branches (indicated by white and yellow arrowheads) of similar calibre arising from a single proximal RCA proper and running in the right AV groove with both branches reaching the crux of heart. [RA: right atrium; RV: right ventricle]

 To the best of our knowledge, the variant encountered in the present case has not been reported previously and is analogous to the widely recognized “dual left anterior descending artery (LAD)” where a short LAD terminates in the proximal part of the anterior interventricular groove and a long LAD, that initially courses outside the anterior interventricular groove, re-enters the anterior interventricular groove in its distal part (Figure 2).^1^ However, in the context of RCA, the term “dual RCA” has traditionally been used to describe the presence of either two parallel RCA arising from separate ostia or two parallel branches of similar calibre arising from a single proximal RCA proper and running in the right AV groove with both branches reaching the crux cordis (Figure 3).^2^ The term “dual RCA” has also been used interchangeably with “duplicated RCA” or “double RCA”.

 In light of recognition of the variant encountered in the present case and existence of a widely recognized term i.e., “dual LAD” to describe an analogous configuration of the LAD, we believe it would be prudent to draw a distinction between “dual” and “duplicated” RCA and reserve the term “dual RCA” to describe a configuration where the RCA proper gives rise to a short RCA that terminates in the midpart of the right AV groove, and a long RCA which initially coursed parallel to the short RCA outside the right AV groove and then re-enters the right AV groove and assumes the distal course of RCA up to the crux cordis. The erstwhile “dual/ duplicated/ double RCA” can be assigned the understandably apt moniker of “duplicated RCA” to avoid confusion in communication of the imaging findings.

## Conclusion

 The distinction between dual RCA and duplicated RCA would bring about a uniformity in the nomenclature of variations of epicardial coronary arteries with both ‘dual RCA’ and ‘dual LAD’ referring to analogous configurations of the RCA and LAD respectively.

## Competing Interests

 The authors declare that they have no conflict of interest.

## Ethical Approval

 Informed consent was obtained from the patient.
